# Supplementary material for: Widespread genetic effect heterogeneity impacts bias and power in nonlinear Mendelian randomization
Source: medRxiv. 2026 Apr 20:2026.04.17.26351133. Preprint. [Version 1] doi: 10.64898/2026.04.17.26351133 (PMC13131742; doi:10.64898/2026.04.17.26351133)
Supplement: Supplement 1 [file NIHPP2026.04.17.26351133v1-supplement-1.pdf]

medRxiv preprint doi: https://doi.org/10.64898/2026.04.17.26351133; this version posted April 20, 2026. The copyright holder for this preprint (which was not certified by peer review) is the author/funder, who has granted medRxiv a license to display the preprint in perpetuity.
It is made available under a CC-BY 4.0 International license .

# Supplemental figures

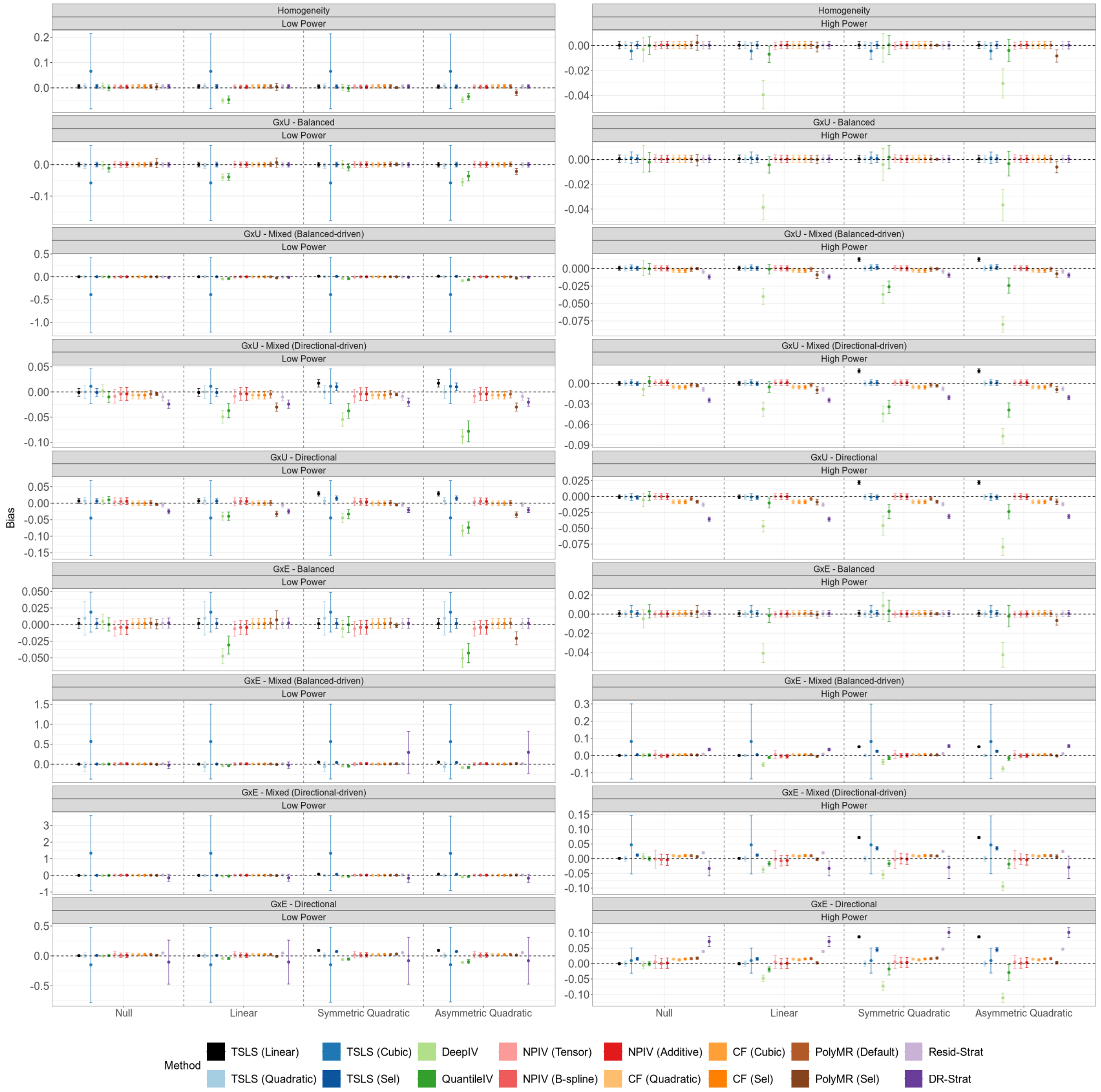


**Figure S1: Average bias of nonlinear MR methods under instrument-exposure effect homogeneity or heterogeneity.** Points indicate the average bias across 100 replicates, and error bars indicate 95% confidence intervals for the mean. Simulation sample size is 50,000 (low power) or 100,000 (high power). We varied the instrument-exposure effect structures across homogeneity, heterogeneity induced by $G \times U$, and heterogeneity induced by $G \times E$, each under balanced ($\pi = 0$), mixed (balanced- ($\pi = 0.33$) or directional-driven ($\pi = 0.66$)), and directional ($\pi = 1$) architectures. TSLS, two-stage least squares; NPIV, NPIV with tensor-product basis (Tensor), generalized B-spline polynomial basis (B-spline), or additive basis (Additive); CF, control function; PolyMR (Default), PolyMR without model selection; PolyMR (Sel), PolyMR with model selection; Resid-Strat, residual stratification; DR-Strat, doubly-ranked stratification.

medRxiv preprint doi: https://doi.org/10.64898/2026.04.17.26351133; this version posted April 20, 2026. The copyright holder for this preprint (which was not certified by peer review) is the author/funder, who has granted medRxiv a license to display the preprint in perpetuity.
It is made available under a CC-BY 4.0 International license .

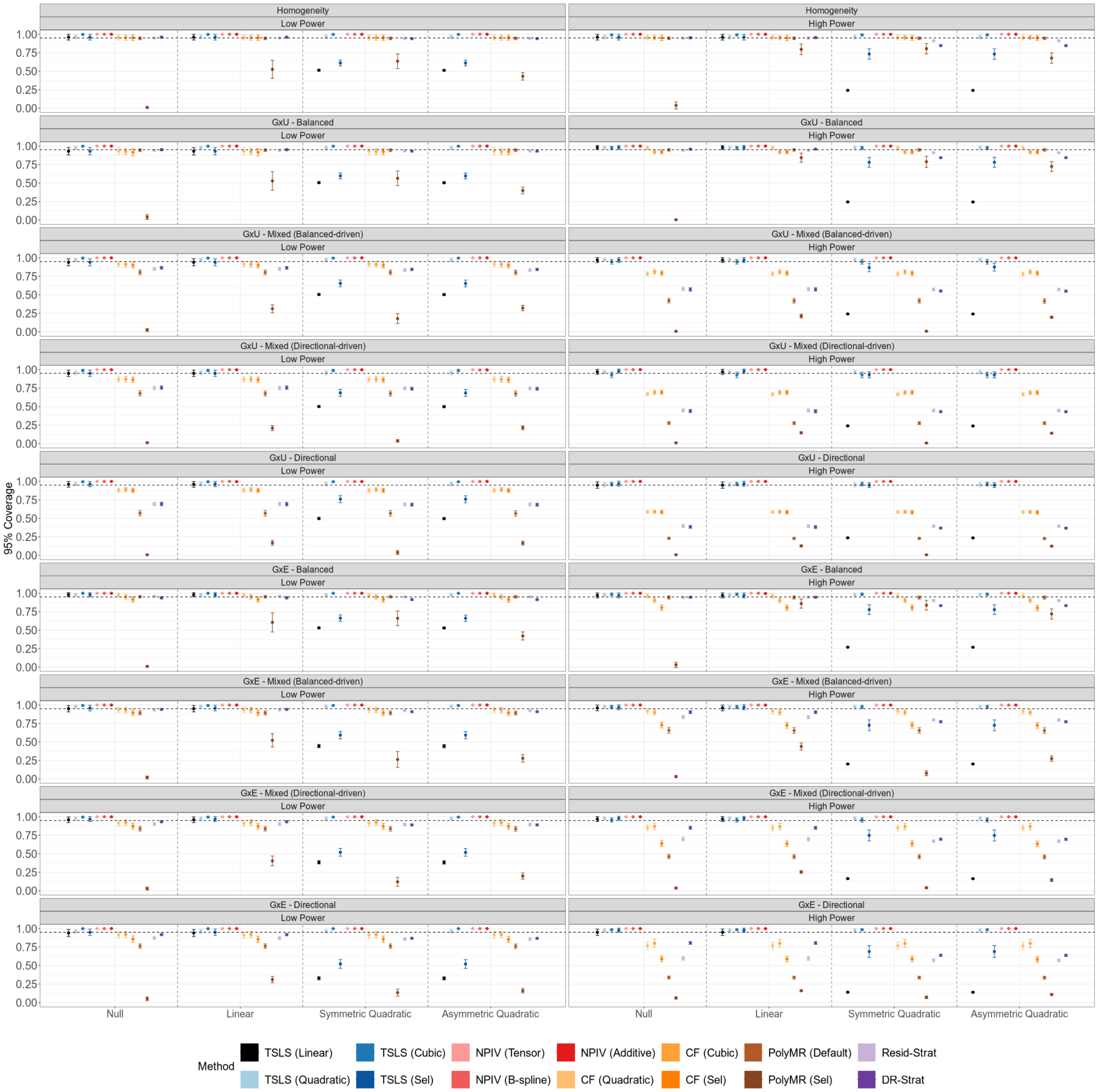


**Figure S2: Average 95% confidence interval coverage of nonlinear MR methods under instrument-exposure effect homogeneity or heterogeneity.** Points indicate the average coverage across 100 replicates, and error bars indicate 95% confidence intervals for the mean. Simulation sample size is 50,000 (low power) or 100,000 (high power). We varied the instrument-exposure effect structures across homogeneity, heterogeneity induced by $G \times U$, and heterogeneity induced by $G \times E$, each under balanced ($\pi = 0$), mixed (balanced- ($\pi = 0.33$) or directional-driven ($\pi = 0.66$)), and directional ($\pi = 1$) architectures. TSLS, two-stage least squares; NPIV, NPIV with tensor-product basis (Tensor), generalized B-spline polynomial basis (B-spline), or additive basis (Additive); CF, control function; PolyMR (Default), PolyMR without model selection; PolyMR (Sel), PolyMR with model selection; Resid-Strat, residual stratification; DR-Strat, doubly-ranked stratification.

medRxiv preprint doi: https://doi.org/10.64898/2026.04.17.26351133; this version posted April 20, 2026. The copyright holder for this preprint (which was not certified by peer review) is the author/funder, who has granted medRxiv a license to display the preprint in perpetuity.
It is made available under a CC-BY 4.0 International license .

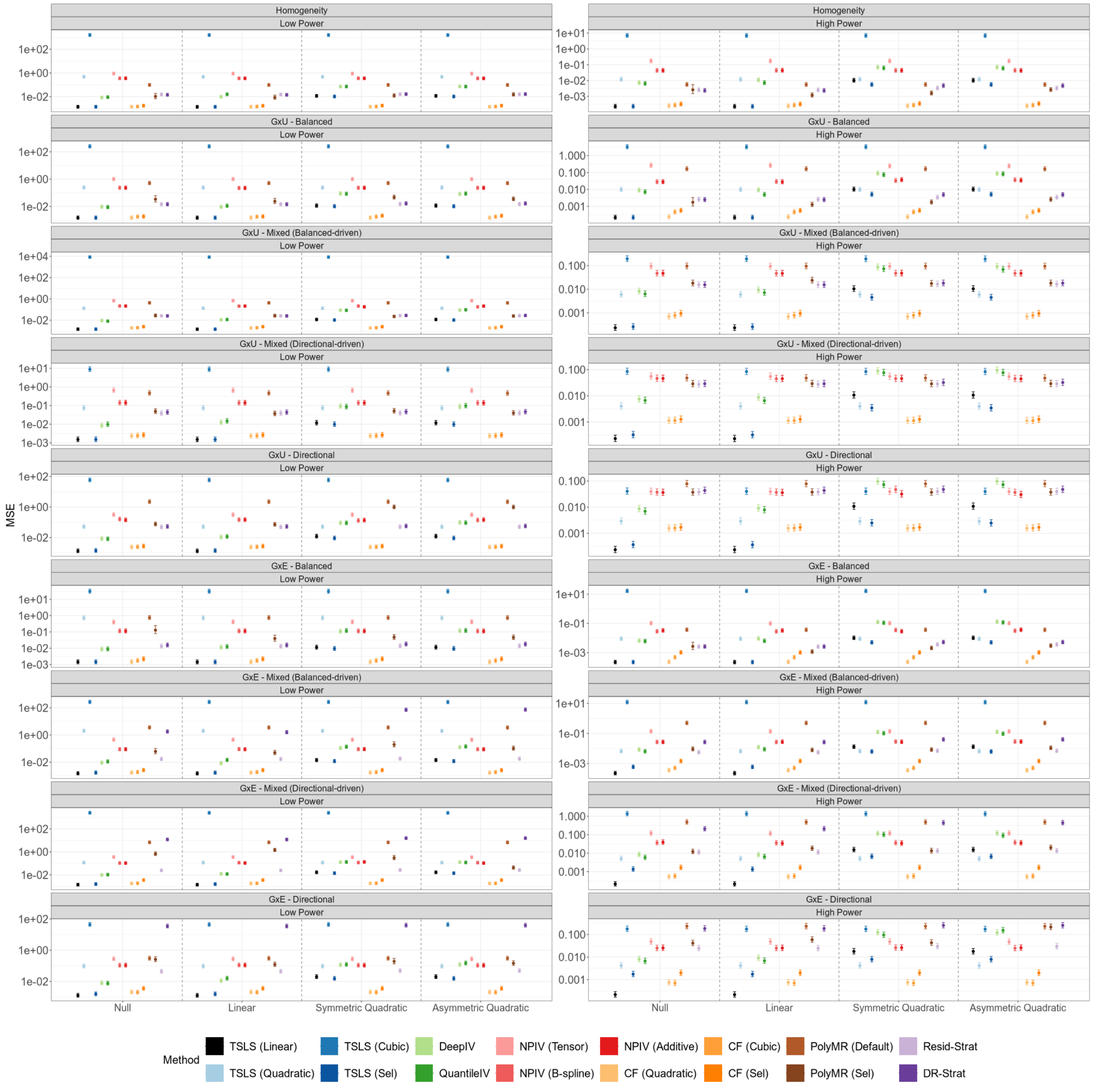


**Figure S3: Average mean squared error (MSE) of nonlinear MR methods under instrument-exposure effect homogeneity or heterogeneity.** Points indicate the average MSE across 100 replicates, and error bars indicate 95% confidence intervals for the mean. Simulation sample size is $50,000$ (low power) or $100,000$ (high power). We varied the instrument-exposure effect structures across homogeneity, heterogeneity induced by $G \times U$, and heterogeneity induced by $G \times E$, each under balanced ($\pi = 0$), mixed (balanced- ($\pi = 0.33$) or directional-driven ($\pi = 0.66$)), and directional ($\pi = 1$) architectures. TSLS, two-stage least squares; NPIV, NPIV with tensor-product basis (Tensor), generalized B-spline polynomial basis (B-spline), or additive basis (Additive); CF, control function; PolyMR (Default), PolyMR without model selection; PolyMR (Sel), PolyMR with model selection; Resid-Strat, residual stratification; DR-Strat, doubly-ranked stratification.

medRxiv preprint doi: https://doi.org/10.64898/2026.04.17.26351133; this version posted April 20, 2026. The copyright holder for this preprint (which was not certified by peer review) is the author/funder, who has granted medRxiv a license to display the preprint in perpetuity.
It is made available under a CC-BY 4.0 International license .

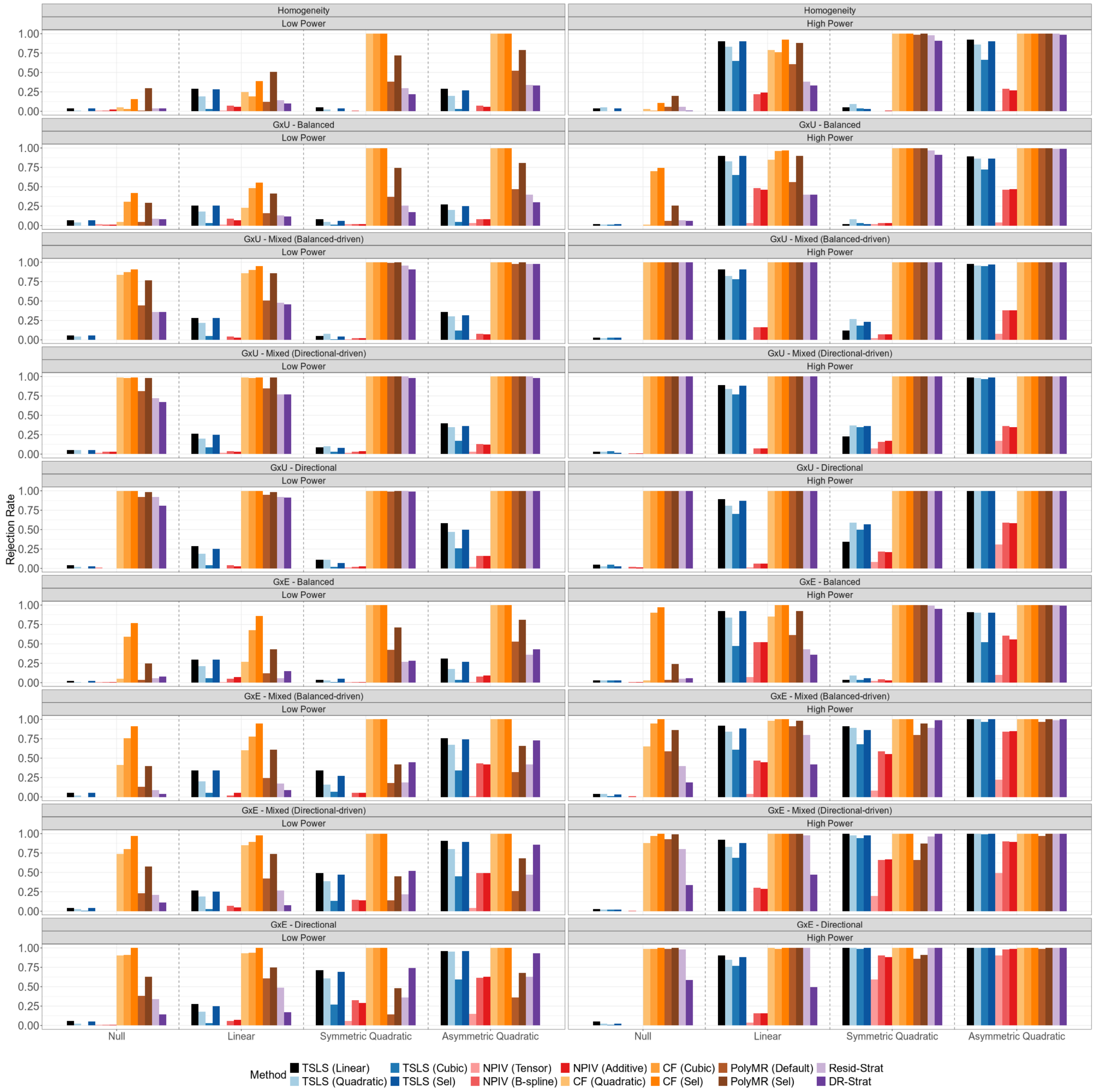


**Figure S4: Rejection rates for overall-effect testing of nonlinear MR methods.** Rejection rates were calculated from $N = 50,000$ or $100,000$ simulated individuals (low- versus high-power settings) per replicate, and summarized across 100 replicates. We varied the instrument-exposure effect structures across homogeneity, heterogeneity induced by $G \times U$, and heterogeneity induced by $G \times E$, each under balanced ($\pi = 0$), mixed (balanced- ($\pi = 0.33$) or directional-driven ($\pi = 0.66$)), and directional ($\pi = 1$) architectures. TSLS, two-stage least squares; NPIV, NPIV with tensor-product basis (Tensor), generalized B-spline polynomial basis (B-spline), or additive basis (Additive); CF, control function; PolyMR (Default), PolyMR without model selection; PolyMR (Sel), PolyMR with model selection; Resid-Strat, residual stratification; DR-Strat, doubly-ranked stratification.

medRxiv preprint doi: https://doi.org/10.64898/2026.04.17.26351133; this version posted April 20, 2026. The copyright holder for this preprint (which was not certified by peer review) is the author/funder, who has granted medRxiv a license to display the preprint in perpetuity.
It is made available under a CC-BY 4.0 International license .

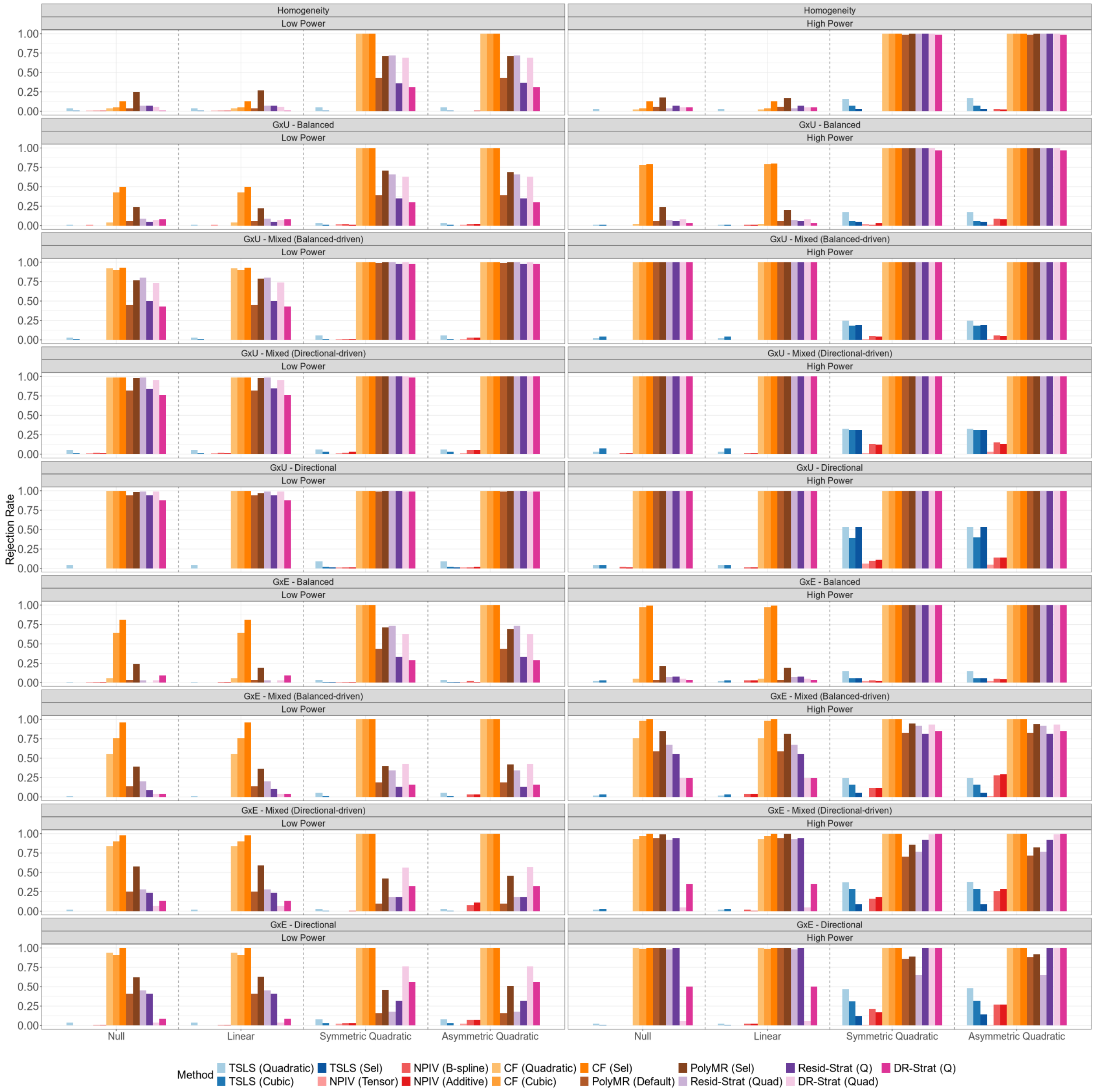


**Figure S5: Rejection rates for nonlinearity testing of nonlinear MR methods.** Rejection rates were calculated from $N = 50,000$ or $100,000$ simulated individuals (low- versus high-power settings) per replicate, and summarized across 100 replicates. We varied the instrument-exposure effect structures across homogeneity, heterogeneity induced by $G \times U$, and heterogeneity induced by $G \times E$, each under balanced ($\pi = 0$), mixed (balanced- ($\pi = 0.33$) or directional-driven ($\pi = 0.66$)), and directional ($\pi = 1$) architectures. TSLS, two-stage least squares; NPIV, NPIV with tensor-product basis (Tensor), generalized B-spline polynomial basis (B-spline), or additive basis (Additive); CF, control function; PolyMR (Default), PolyMR without model selection; PolyMR (Sel), PolyMR with model selection; Resid-Strat, residual stratification; DR-Strat, doubly-ranked stratification; Quad, quadratic test; Q, Cochran's Q test.

medRxiv preprint doi: https://doi.org/10.64898/2026.04.17.26351133; this version posted April 20, 2026. The copyright holder for this preprint (which was not certified by peer review) is the author/funder, who has granted medRxiv a license to display the preprint in perpetuity.
It is made available under a CC-BY 4.0 International license .

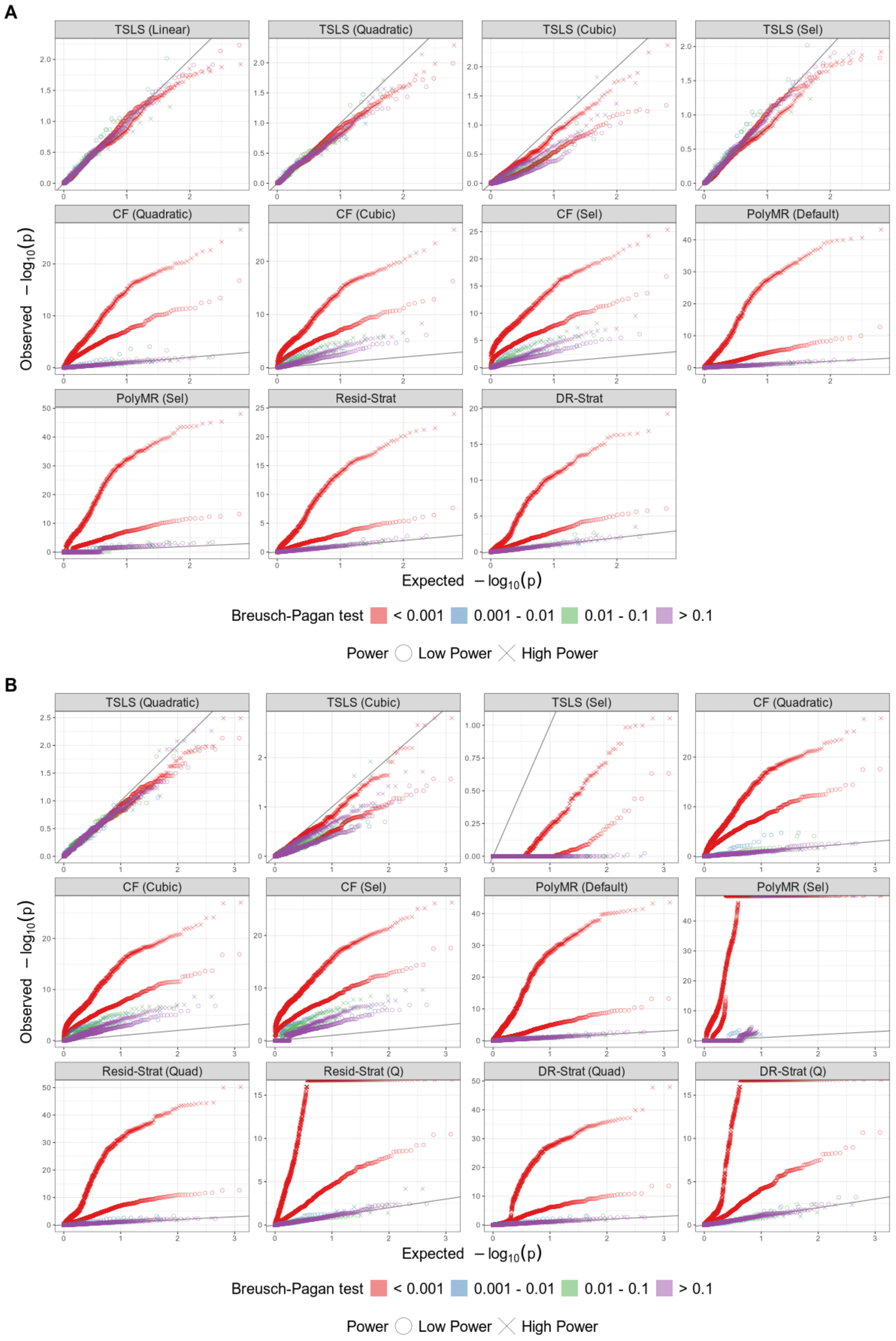


**Figure S6: Quantile–Quantile plots for overall-effect and nonlinearity testing of nonlinear MR methods.** (A) Overall-effect testing, evaluated under the null hypothesis of no causal effect. (B) Nonlinearity testing, evaluated under the null hypothesis of no causal effect or a linear effect. P-values were evaluated from $N = 50,000$ or $100,000$ simulated individuals (low- versus high-power settings), and pooled across all homogeneity and heterogeneity scenarios over 100 replicates. TSLS, two-stage least squares; CF, control function; PolyMR (Default), PolyMR without model selection; PolyMR (Sel), PolyMR with model selection; Resid-Strat, residual stratification; DR-Strat, doubly-ranked stratification; Quad, quadratic test; Q, Cochran's Q test.

medRxiv preprint doi: https://doi.org/10.64898/2026.04.17.26351133; this version posted April 20, 2026. The copyright holder for this preprint (which was not certified by peer review) is the author/funder, who has granted medRxiv a license to display the preprint in perpetuity.
It is made available under a CC-BY 4.0 International license .

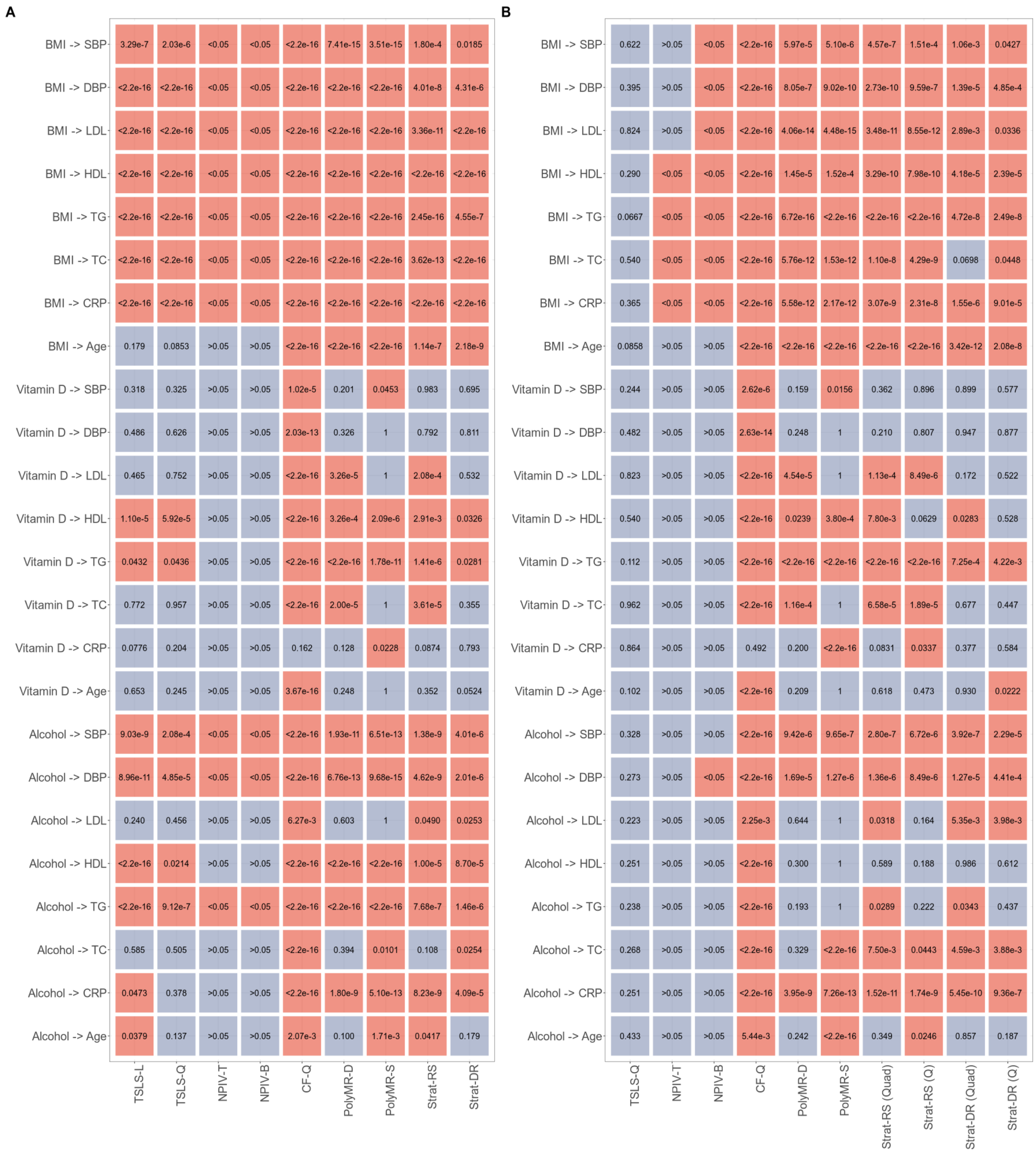


**Figure S7: Mendelian randomization results for overall-effect and nonlinearity inference of BMI, vitamin D, and alcohol consumption on blood pressure, lipid, C-reactive protein, and age in UK Biobank.** (A) Overall-effect testing. (B) Nonlinearity testing. Numbers denote p-values for the corresponding test; red indicates nominal significance at $\alpha = 0.05$ and blue indicates non-significant results. TSLS-L, linear two-stage least squares; TSLS-Q, quadratic two-stage least squares; NPIV-T, NPIV with tensor-product basis; NPIV-B, NPIV with generalized B-spline polynomial basis; CF-Q, quadratic control function; PolyMR-D, PolyMR without model selection (default); PolyMR-S, PolyMR with model selection; Strat-RS, residual stratification; Strat-DR, doubly-ranked stratification; Quad, quadratic test; Q, Cochran's Q test; SBP, systolic blood pressure; DBP, diastolic blood pressure; LDL, low-density lipoprotein cholesterol; HDL, high-density lipoprotein cholesterol; TG, triglycerides; TC, total cholesterol; CRP, C-reactive protein.

medRxiv preprint doi: https://doi.org/10.64898/2026.04.17.26351133; this version posted April 20, 2026. The copyright holder for this preprint (which was not certified by peer review) is the author/funder, who has granted medRxiv a license to display the preprint in perpetuity.
It is made available under a CC-BY 4.0 International license .

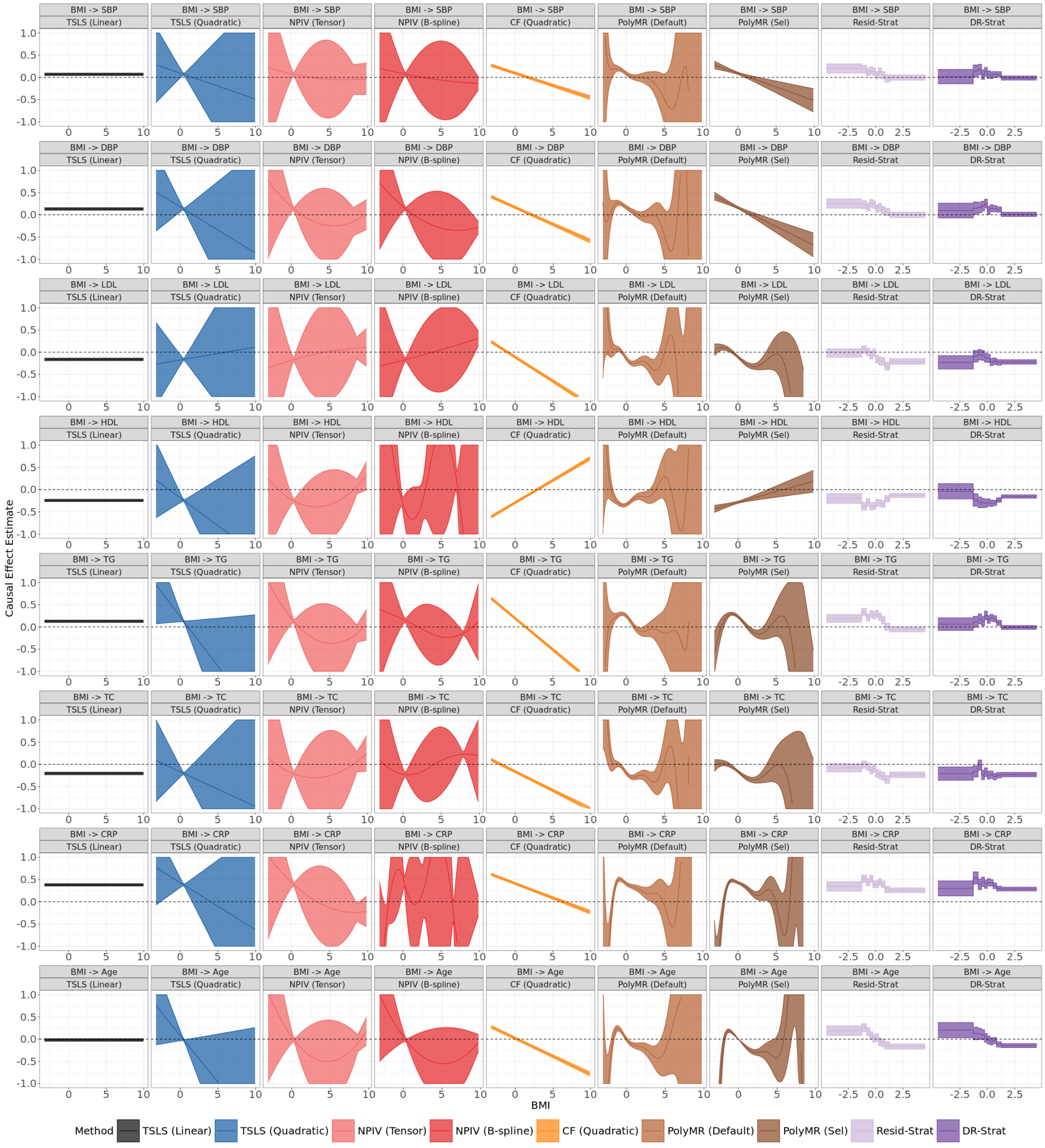


**Figure S8: Estimated causal shapes of BMI on blood pressure, lipid, C-reactive protein, and age in UK Biobank.** Shaded regions indicate 95% confidence bands. The x-axis denotes standardized BMI, except for stratification-based methods, where it represents rank-based inverse-normal-transformed post-stratification BMI. TSLS, two-stage least squares; NPIV, NPIV with tensor-product basis (Tensor), generalized B-spline polynomial basis (B-spline); CF, control function; PolyMR (Default), PolyMR without model selection; PolyMR (Sel), PolyMR with model selection; Resid-Strat, residual stratification; DR-Strat, doubly-ranked stratification; SBP, systolic blood pressure; DBP, diastolic blood pressure; LDL, low-density lipoprotein cholesterol; HDL, high-density lipoprotein cholesterol; TG, triglycerides; TC, total cholesterol; CRP, C-reactive protein.

medRxiv preprint doi: https://doi.org/10.64898/2026.04.17.26351133; this version posted April 20, 2026. The copyright holder for this preprint (which was not certified by peer review) is the author/funder, who has granted medRxiv a license to display the preprint in perpetuity.
It is made available under a CC-BY 4.0 International license .

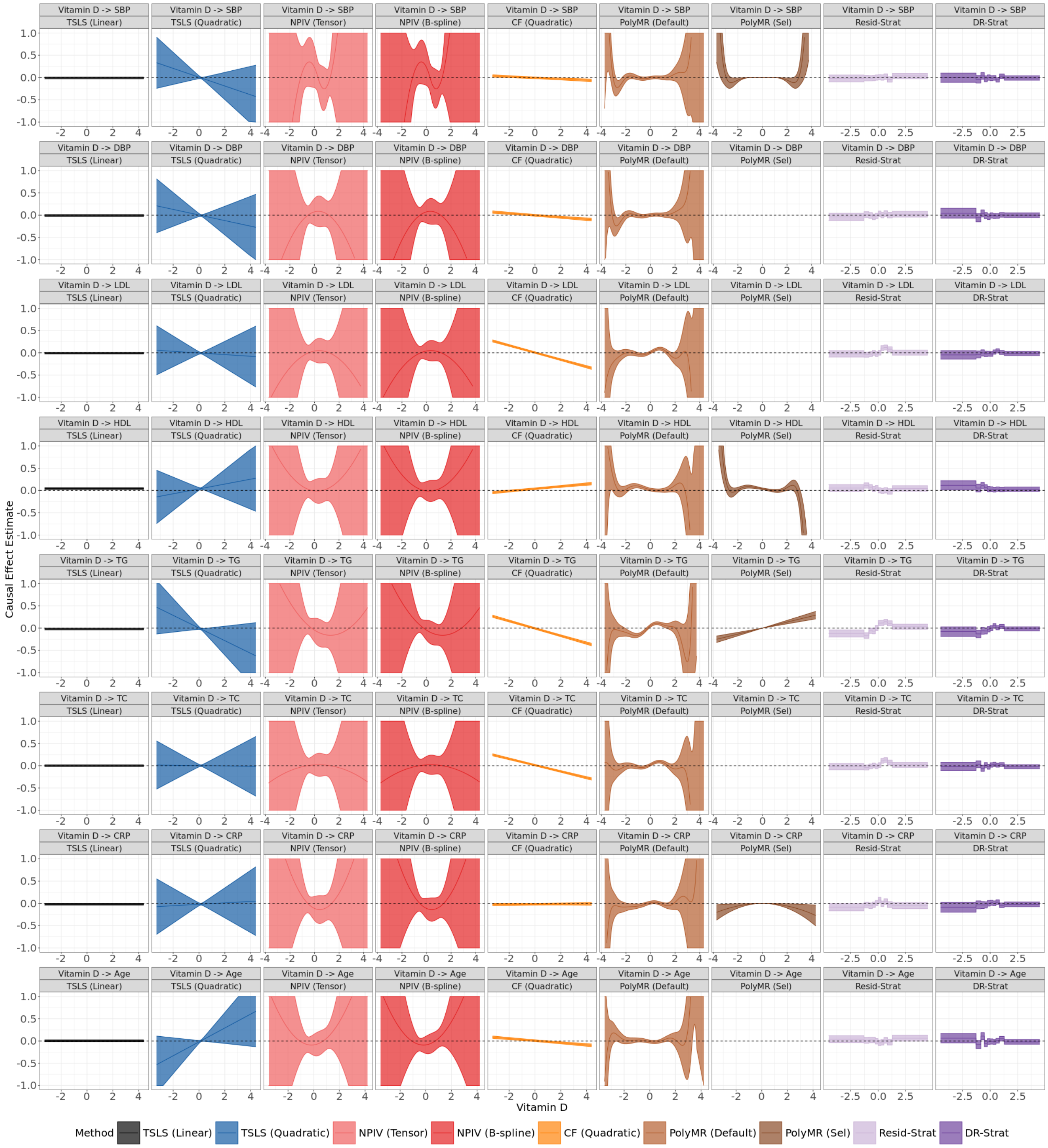


**Figure S9: Estimated causal shapes of vitamin D on blood pressure, lipid, C-reactive protein, and age in UK Biobank.** Shaded regions indicate 95% confidence bands. The x-axis denotes standardized vitamin D (natural-log scale), except for stratification-based methods, where it represents rank-based inverse-normal-transformed post-stratification vitamin D (natural-log scale). TSLS, two-stage least squares; NPIV, NPIV with tensor-product basis (Tensor), generalized B-spline polynomial basis (B-spline); CF, control function; PolyMR (Default), PolyMR without model selection; PolyMR (Sel), PolyMR with model selection; Resid-Strat, residual stratification; DR-Strat, doubly-ranked stratification; SBP, systolic blood pressure; DBP, diastolic blood pressure; LDL, low-density lipoprotein cholesterol; HDL, high-density lipoprotein cholesterol; TG, triglycerides; TC, total cholesterol; CRP, C-reactive protein.

medRxiv preprint doi: https://doi.org/10.64898/2026.04.17.26351133; this version posted April 20, 2026. The copyright holder for this preprint (which was not certified by peer review) is the author/funder, who has granted medRxiv a license to display the preprint in perpetuity.
It is made available under a CC-BY 4.0 International license .

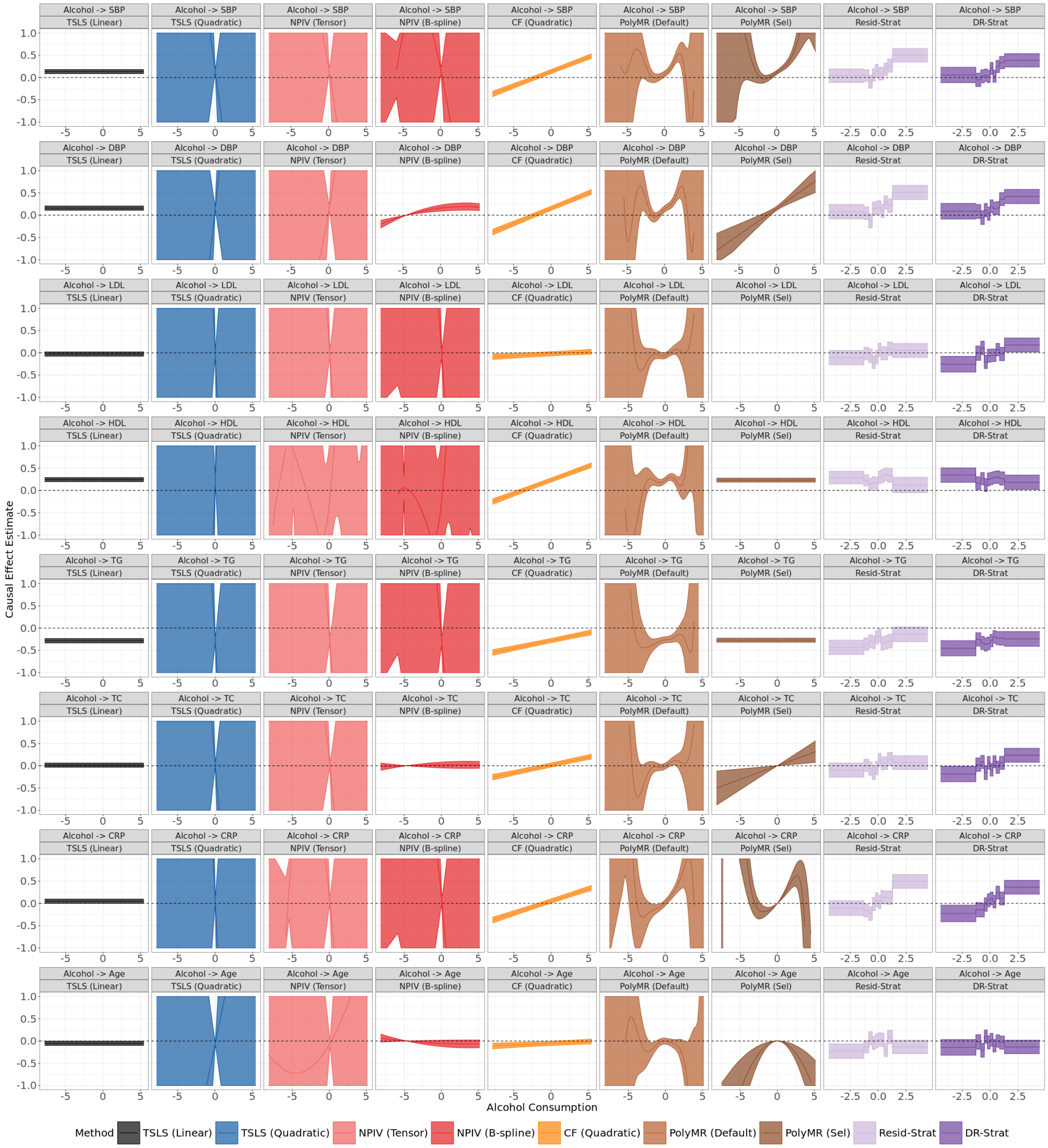


**Figure S10: Estimated causal shapes of alcohol consumption on blood pressure, lipid, C-reactive protein, and age in UK Biobank.** Shaded regions indicate 95% confidence bands. The x-axis denotes standardized alcohol consumption (natural-log scale), except for stratification-based methods, where it represents rank-based inverse-normal-transformed post-stratification alcohol consumption (natural-log scale). TSLS, two-stage least squares; NPIV, NPIV with tensor-product basis (Tensor), generalized B-spline polynomial basis (B-spline); CF, control function; PolyMR (Default), PolyMR without model selection; PolyMR (Sel), PolyMR with model selection; Resid-Strat, residual stratification; DR-Strat, doubly-ranked stratification; SBP, systolic blood pressure; DBP, diastolic blood pressure; LDL, low-density lipoprotein cholesterol; HDL, high-density lipoprotein cholesterol; TG, triglycerides; TC, total cholesterol; CRP, C-reactive protein.

medRxiv preprint doi: https://doi.org/10.64898/2026.04.17.26351133; this version posted April 20, 2026. The copyright holder for this preprint (which was not certified by peer review) is the author/funder, who has granted medRxiv a license to display the preprint in perpetuity.
It is made available under a CC-BY 4.0 International license .

# Supplemental notes

## Note S1: Details of nonlinear MR method implementation

We implemented Deep IV and Quantile IV using the `ml-mr` Python package, with the `--exposure-network-type gaussian_net` option for Deep IV and the `--n-quantiles 10` option for Quantile IV. NPIV was implemented using the `npiv` R package, with three basis specifications: (1) tensor-product basis (default), (2) generalized B-spline polynomial basis (`basis = "glp"`), and (3) additive basis (`basis = "additive"`). PolyMR was implemented using the `PolyMR` R package, both without model selection (default) and with model selection (`p_thr_drop = NULL`). Residual stratification and doubly-ranked stratification were implemented using the `SUMnlmr` R package.
